# Microfluidics-Based Plasmonic Biosensing System Based on Patterned Plasmonic Nanostructure Arrays

**DOI:** 10.3390/mi12070826

**Published:** 2021-07-14

**Authors:** Yanting Liu, Xuming Zhang

**Affiliations:** Department of Applied Physics, Hong Kong Polytechnic University, Hong Kong; yantinliu2-c@my.cityu.edu.hk

**Keywords:** microfluidics, plasmonics, biosensors, surface plasmon resonance, plasmonic nanostructure arrays

## Abstract

This review aims to summarize the recent advances and progress of plasmonic biosensors based on patterned plasmonic nanostructure arrays that are integrated with microfluidic chips for various biomedical detection applications. The plasmonic biosensors have made rapid progress in miniaturization sensors with greatly enhanced performance through the continuous advances in plasmon resonance techniques such as surface plasmon resonance (SPR) and localized SPR (LSPR)-based refractive index sensing, SPR imaging (SPRi), and surface-enhanced Raman scattering (SERS). Meanwhile, microfluidic integration promotes multiplexing opportunities for the plasmonic biosensors in the simultaneous detection of multiple analytes. Particularly, different types of microfluidic-integrated plasmonic biosensor systems based on versatile patterned plasmonic nanostructured arrays were reviewed comprehensively, including their methods and relevant typical works. The microfluidics-based plasmonic biosensors provide a high-throughput platform for the biochemical molecular analysis with the advantages such as ultra-high sensitivity, label-free, and real time performance; thus, they continue to benefit the existing and emerging applications of biomedical studies, chemical analyses, and point-of-care diagnostics.

## 1. Introduction

Microfluidics refers to systems for the control and manipulation of fluids at small scale, which can process or manipulate small amounts of fluids (10^−9^ to 10^−18^ L) at sub-millimeter dimension channels. These microfluidics systems can facilitate the development of micro total analysis systems (µTAS) or lab-on-a-chip (LoC) systems. Such kinds microfluidic systems can automate and include all the necessary steps for the chemical analysis of a sample, such as sample transport, chemical reactions, and detection. It can also enhance transport and realize high-throughput processing of the flow conditions. Furthermore, the microfluidic system can reduce the dosages of samples and reagents (down to nanoliter scale) while increasing the mixing rate of different reagents [1]. Microfluidic devices and systems have been demonstrated to be well suitable for manipulating biomolecules, cells, or small-scale particles, which have made substantial contributions to biology and medical research over the past several decades [2,3]. The compelling advantages in microfluidic approaches, such as high-throughput processing ability with automatic control, the sample volume substantially with less dosage, and scalability for screening applications, have superior properties over traditional assays used in biomedical analysis and diagnostics [4,5]. In recent years, many microfluidic studies have been dedicated to developing various methods with enhanced capabilities to replace traditional macroscale assays [6,7]. Still, addressing such formidable challenges requires advanced techniques in numerous disciplines and interdisciplinary areas, such as sensing, micro pumping techniques, and cell culture or manipulation platforms [8,9,10,11].

The microfluidic system is a miniature operating platform, which greatly reduces the complexity and consumption of time of assay. Meanwhile, the microfluidic devices are easy to be combined with other sensing detection techniques for assay because of its feasible principle and simple construction. Microfluidic devices integrated with on-chip sensors have been developed for various biological and biomedical assays due to their great potential for clinic practice applications [12,13]. Biosensors are analytical devices that are integrated within a physicochemical transducer or transducing microsystems to incorporate biological recognition molecules or elements by immobilization [14,15]. Biosensors can be used for sensing a wide range of biomolecules including enzymes, aptamers, immune proteins such as antibodies and antigens, nucleic acids such as DNA and RNA, and microorganisms such as bacteria and pathogens. Furthermore, cancer or tumor cells can also be detected by biosensors that can even achieve single-cell resolution [12]. The biosensor can detect the target biomolecules with high sensitivity and have the ability to perform multiplex analysis. Therefore, the biosensing technique has been considered as a powerful analytical tool for disease detection, medical diagnosis, and treatment at an early stage in the biomedical field. The biosensor is capable of converting biological or chemical molecules’ responses into different signals such as electrical chemical, optical, color or mass change, and so on. The biosensor can provide analytical assay through biorecognition components with different types of integrated transducer. Hence, biosensors can be cataloged as electrochemical, mechanical, and optical biosensors [16,17]. The electrochemical biosensors can measure electrochemical signal changes caused by the biochemical interaction between molecules with simple manufacturing methods [18,19]. Meanwhile, for mechanical biosensors, the changes of mass on the surface of the transducer caused by the biochemical interaction are measured for signal detection [20,21].

Among different kinds of biosensors, the optical biosensor is one of the most commonly reported biosensors. By integrating a biorecognition sensing part on the optical signal detection system, it can measure the changes in light when binding analyte molecules [22,23]. Generally, optical biosensors can directly detect biochemical interaction through various optical techniques such as optical fibers, optical waveguides, and surface plasmon resonance (SPR) [24,25]. It can measure the variations of an optical transducer including intensity, wavelength, refractive index, and polarization properties of light [26]. The optical biosensor can also indirectly detect analytes through optically labeled probes, such as fluorescence and luminescence [27,28,29]. Furthermore, the optical biosensor can accomplish multiplex monitoring and analysis of bioreceptor molecules by measuring different signals from multiple analytes [30]. Compared with other biosensing methods, optical biosensors exhibit unique advantages such as being non-destructive with less interference, having a low detection limit with high sensitivity, and having low background noise and fast signal monitoring, which have been comprehensively reviewed in the literature [23,31,32]. As for various optical biosensors, the plasmonic sensor represents the most advanced, well-established, and rapidly developed biosensing technology [33,34]. Plasmonic biosensors focus on exploiting the unique electromagnetic waves-surface plasmon-polaritons-to probe interactions, where bio/chem molecular recognition components are immobilized on the surface of the sensor [35,36].

**Figure 1:**
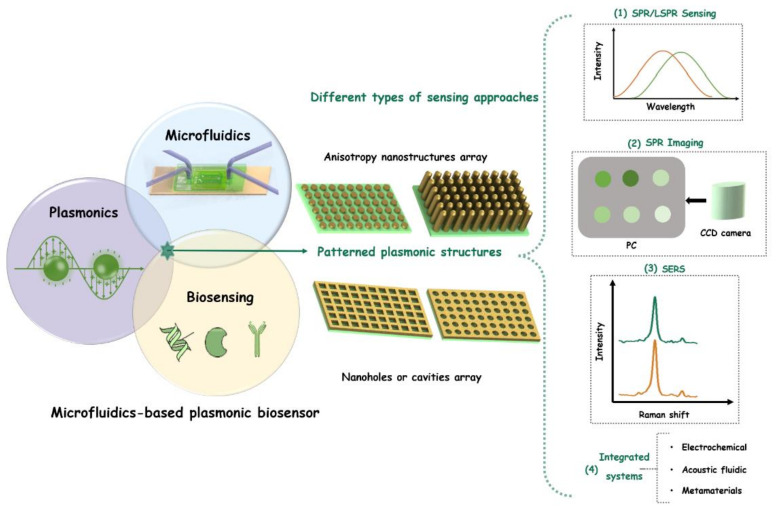
Schematic illustration of microfluidic-integrated plasmonic biosensor system based on patterned plasmonic arrays with different types of sensing approaches.

As mentioned above, benefiting from the ease of multiplexing and integration of a microfluidic chip, microfluidics-based biosensing detection techniques could give rise to new opportunities for potential applications in biological and bio-medical fields [37,38]. The advantages of microfluidic chip integration including high-throughput, miniaturization, and low-cost features that would improve the performance of the detection systems [39,40]. It can promote ultrasensitive analysis of plasmonic biosensing systems for the simultaneous detection of analytes with multiplex processing ability [41,42,43]. In this review, different types of microfluidic-integrated plasmonic biosensors are summarized and represented. It gives some examples of plasmonic biosensors integrated with microfluidics technology in the literature in the last 15 years and states the advantages of merging microfluidic and plasmonic technologies for biosensing applications. The roadmap for this review on the plasmonic biosensor integrated with microfluidics platform technology is illustrated in Figure 1. Particularly, this review focuses on versatile patterned plasmonic nanostructured arrays based on plasmonic biosensing in microfluidic systems. It will be introduced in detail in the following sections including (i) plasmonic biosensors; (ii) plasmonic biosensors based on patterned plasmonic nanostructured arrays; and (iii) different types of sensing approaches for microfluidic-integrated patterned plasmonic nanostructured arrays-based biosensors platforms. We aim at illustrating the versatility and perspective of such integrated microfluidics-based plasmonic biosensing techniques for numerous areas in biological, biomedical applications, and promising clinical biosensing for point-of-care diagnostics in the future.

## 2. Plasmonic Biosensors

### 2.1. Principle of Plasmons

The basic principle of plasmonic is well-known and has been extensively reviewed [44,45]. Plasmons are collective oscillations of the free electrons, which exist at the bulk and surface of the metal and in the neighborhood of nanoparticles. It can be classified as bulk, surface, and localized surface (nanoparticle) plasmons, respectively [46]. In the case of surface plasmon resonances (SPRs) at the surface of a metal, plasmonic modes can be excited along with the metal–dielectric interface and exhibit an evanescent field that could penetrate the surrounding media [47]. While for the case of localized surface plasmon, a surface plasmon is confined and excited on sub-wavelength-sized metal nanoparticles with a specific frequency known as localized surface plasmon resonance (LSPR) [48]. Typically, the SPR can be used as a refractometric sensing platform due to the high sensitivity of the evanescent field to refractive index (RI) changes when close to the metal surface [49]. Similar to the SPR, the LSPR of plasmonic nanostructures is also sensitive to changes in the local dielectric environment, which can measure the LSPR wavelength-shift of nanoparticles [50].

### 2.2. Conventional SPR Biosensing

**Figure 2:**
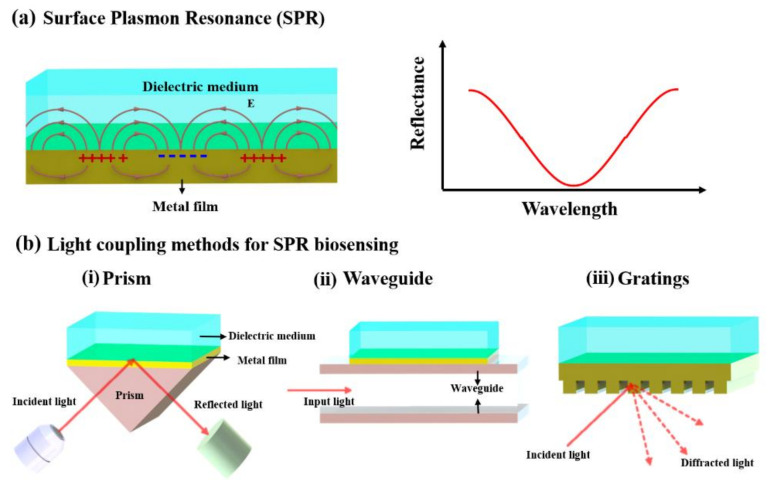
Schematic representations of (**a**) surface plasmon resonance (SPR) at a flat metal–dielectric interface. (**b**) The excitation of surface plasmon–polariton by different light coupling methods for SPR biosensing, including (**i**) by prism coupling-based Kretschmann configuration, (**ii**) by the optical wave-guide coupling, and (**iii**) by light diffraction grating coupling.

Plasmonic sensors have attracted increasing attention since the first SPR-based refractometric sensors were developed by Nylander and Liedberg et al. in 1983 [51]. The SPR biosensor has become the most commonly used and commercialized optical sensor. It can be utilized for different types of bio/chem molecules interactions detection, which show excellent potential application in biological, biomedicine, and diagnostics-related fields [52]. For a typical SPR biosensor, it is based on the propagation of surface plasmon polaritons resonance, where electromagnetic waves could occur at the interface between a flat metal film and dielectric, as shown in Figure 2a. The SPR usually presents in transverse-magnetic (TM) mode and can be characterized by its propagation vector and electromagnetic field distribution. The propagation vector of the SPR (*β_SPR_*) can be expressed as:

(1) $$\beta_{SPR}=\frac{\omega }{c}\sqrt{\frac{\varepsilon_{m}\varepsilon_{d}}{\varepsilon_{m}+\varepsilon_{d}}}$$

where *ω* is the angular frequency, *c* is the speed of light in a vacuum, so $\frac{\omega }{c}$ is the wave vector of the light in a vacuum. *ε_m_* and *ε_d_* are dielectric functions of the dielectric and metal, respectively [41,51]. For the SPR excitation and propagation, the real part of the *ε_m_* must be negative, n and its absolute value is smaller than *ε_d_*. This condition can be fulfilled for several noble metals, especially for the most commonly employed metal gold (Au)-based SPR biosensors [51,52]. Furthermore, this equation can explain the main principle of refractometric sensing platforms. The bio/chem molecules’ interactions usually take place at the vicinity of the interface between metal and dielectric, which could induce refractive index (RI) changes leading to the change of the SPR propagation condition. The above changes can be detected in real time and transform into measurable and quantifiable signal in label-free ways [49].

Generally, in SPR sensors, surface plasmon–polariton can be excited by a light wave and can be measured by the effect of this interaction through the properties of the light wave. Therefore, the changes in the propagation vector of the surface plasmon–polariton can be determined by the above measurements. However, the propagation vector of the SPR (*β_SPR_*) is considerably larger than the wavenumber of the light wave in the dielectric medium, so surface plasmons cannot be excited by direct illumination [41,49]. Excitation of a surface plasmon-polariton by light can occur when the SPR matches with the component of the wave vector of light, which is parallel to the metal surface. The excitation of SPR can be achieved through enhancing the incoming light by several different approaches including (i) prism coupling [53], (ii) waveguide coupling [54], and (iii) grating coupling [51] (Figure 2b).

### 2.3. LSPR Biosensing

In recent decades, with the excellent development of nanotechnology, enormous progress and new methods have been made and provided to fabricate and assemble nanomaterials. Consequently, nanotechnology has been introduced and applied to various fields including biosensing-based fields [55]. Nanomaterial-based biosensors have been widely utilized in biosensing detection. Especially, numerous research studies have focused on noble metal nanomaterials due to their unique optical properties in the nano-plasmonic field [56,57]. The plasmonic nanoparticles have several advantages including large surface energy, high stability, superior biocompatibility, and strong signals amplification, which have made them excellent for biosensor application. Hence, the nano-plasmonic studies have led to the development of LSPR-based sensors, such as the LSPR refractometric biosensors [58,59]. Due to unique nanoscale plasmonic components, LSPR biosensors can realize ultrasensitive and selectivity detection [60,61].

**Figure 3:**
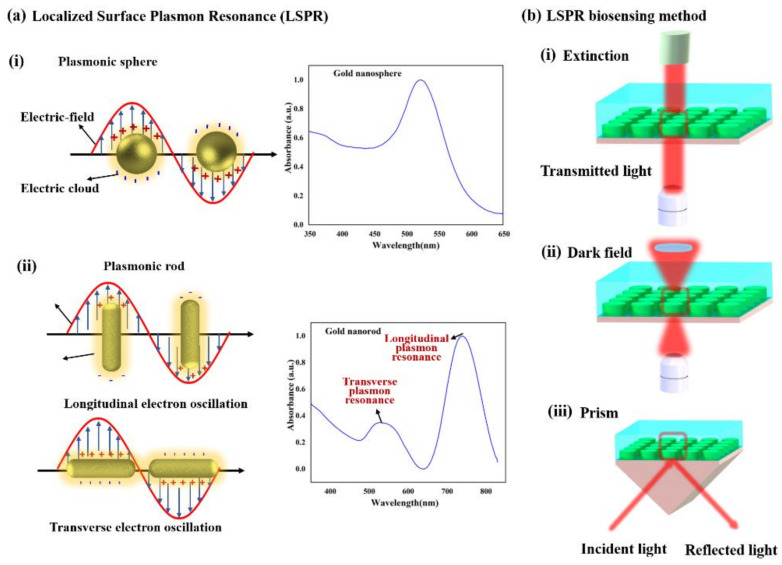
Schematic representations of (**a**) the localized surface plasmon resonance (LSPR) of (**i**) plasmonic nanosphere and (**ii**) plasmonic nanorod positioned in a static electric field. (**b**) LSPR biosensing methods, including (**i**) extinction, (**ii**) dark-field, and (**iii**) prism coupler on the plasmonic nanostructured surface.

Generally, LSPR originates from collectively or non-propagating oscillation of the conduction electrons due to the interaction between metal nanostructures and electromagnetic (EM) field or incident light [62]. This strong extinction of light could lead to some of the incident photons being absorbed and some being scattered in different directions, which can cause the accumulation of polarization charges on the surface of the metallic nanostructures [63]. When the LSPR of the plasmonic nanostructure is excited by incident light, the absorption, scattering, and EM field close to the nanostructure surface can be greatly enhanced [64]. The LSPR properties of the plasmonic nanostructure can be regulated by the morphology, size, component, and distance between adjacent nanostructures [65]. As shown in Figure 3a(i), the metal nanosphere possesses a single dipole of surface plasmon mode and displays only one main peak in the optical spectrum during electrons’ oscillation [66]. Compared with a metal nanosphere, for an anisotropic metal nanostructure, especially for nanorod, the surface plasmons split into two distinct modes due to the high aspect ratio and surface curvature of geometry [62]. The metal nanorod exhibits strong polarization-dependent optical spectra, and it has two principal plasmon absorption bands, corresponding to the transverse plasmon resonance (TPR) and the longitudinal plasmon resonance (LPR) modes, as shown in Figure 3a(ii) [67]. Hence, LSPR properties of the plasmonic nanostructure can be tailored and tuned from ultraviolet (UV), visible (VIS), to near-infrared (NIR) regions in the optical spectrum by varying the chemical and physical properties of the plasmonic nanostructures [68]. This unique LSPR-dependent property of plasmonic nanostructures can be enormously useful for many applications, particularly for biosensing [69].

Analogous to SPR biosensing, the wavelength of the LSPR can be characterized by the sum of light absorption and scattering called extinction [50]. Its wavelength peak is extremely sensitive to the RI of surrounding media and can be spectrally monitored [70]. The binding of an analyte on the surface of the plasmonic nanostructures could result in the RI change, consequently causing a shift in the extinction peak wavelength, *λ_max_*. This shift in *λ_max_* is given by the following equation [71]:

(2) $$∆\lambda_{max\;}≅m∆n\left[1-exp\left(\frac{-2d}{l_{d}}\right)\right]$$

where *m* is the sensitivity factor, Δ*n* is the change in RI, d is the thickness of the effective adsorbate layer, and *l_d_* is the decay length of the EM field. The extinction is maximized through optimizing the intrinsic parameters of plasmonic nanostructures, such as shape, size, component elements, and structure orientation [72]. Such advancement of LSPR biosensing is capable of gaining opportunities to integrate the conventional coupling mode of the light with miniaturization, multiplexing, and high-resolution detection and analysis [60]. The plasmonic sensing component can be integrated down with nanoscale-size structures, and the LSPR effect can generate sufficiently through simple transmission or reflection configurations [73]. The LSPR biosensing methods can be classified into the following approach as shown in Figure 3b: (i) extinction, (ii) dark field, and (iii) prism on a metal nanostructured surface [71,74]. The extinction mode is the most straightforward method by measuring the extinction spectrum of the LSPR induced by the specific binding of the analyte to the surface of the patterned plasmonic nanostructure arrays [75]. Meanwhile, the dark-field light-scattering configuration method is an extremely powerful measurement tool when involving the analytes in small regions or even single nanoparticles. The dark-field mode allows adjusting the spot size of the plasmonic nanostructured region via precisely controlling over a high-numerical aperture condenser of both light-illuminating and light-collecting lenses of the sensing system [75,76]. On the contrary, the prism coupler in plasmonic nanostructure array mode has no numerical restriction on the objective aperture by placing surface total internal reflection geometry, which is similar to the Kretschmann configuration SPR sensing system setup [77].

## 3. Plasmonic Biosensors Based on Patterned Metallic Nanostructure Arrays

**Figure 4:**
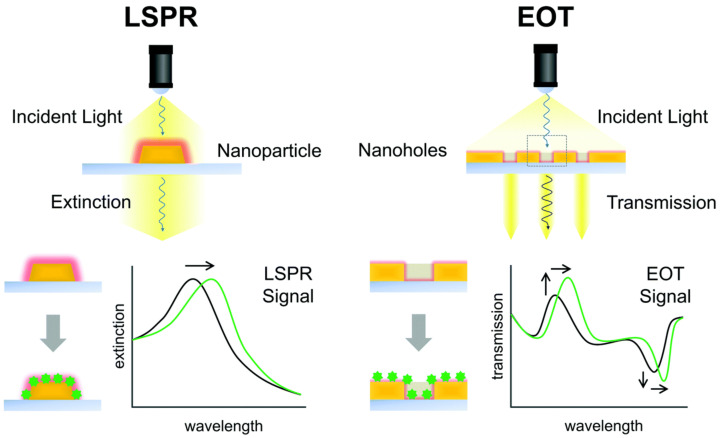
Schematic illustration of principles of the plasmonic metallic nanostructure-based biosensors. The sensing strategies for a nanoparticle structures array is based on LSPR extinction spectrum sensing, while for nanohole or cavity array, it is based through an extraordinary optical transmission (EOT) spectrum. Reproduced with permission from [81]. Copyright 2017 Royal Society of Chemistry.

Significant innovations have led to advantages in low-cost and large-area nanofabrication methods. The usage of various plasmonic metallic nanostructures and integration with microfluidic systems have boosted the quick development of plasmonic biosensors in recent years [78,79,80]. In this review, we focus mainly on patterned plasmonic arrays-based plasmonic biosensors including nanoparticle structures array and nanohole or cavity array substrates. Generally, the patterned plasmonic arrays have two main sensing strategies for nanoparticle structures array and nanohole or cavity array substrates, as shown in Figure 4, which are commonly employed for plasmonic biosensor measurements [81]. The above two types of plasmonic arrays have different optical responses of plasmons for their sensing strategies. The extinction of LSPR sensing is applied for nanoparticle array sensing strategy, while extraordinary optical transmission (EOT) is applied for nanohole or cavity array one [82,83,84]. On the other hand, EOT arises from the interaction of nanohole or cavity with incident light with nanohole via a long-range periodic or short-range ordering manner [81].

### 3.1. Nanoparticle Arrays

The plasmonic biosensors using nanoparticle arrays have superiority when compared with other nano-plasmonic biosensors [85]. The use of nanostructured surfaces instead of colloidal nanoparticles solutions can avoid the cluster and agglomeration phenomenon. Furthermore, it offers a better reproducibility of the analysis by controlling the LSPR of the interaction of the nanostructured array. In addition, such kinds of anisotropic patterned nanoparticle array-based biosensors can integrate with microfluidic technology for multiplexed and ultrasensitive analysis [86,87,88,89,90].

**Figure 5:**
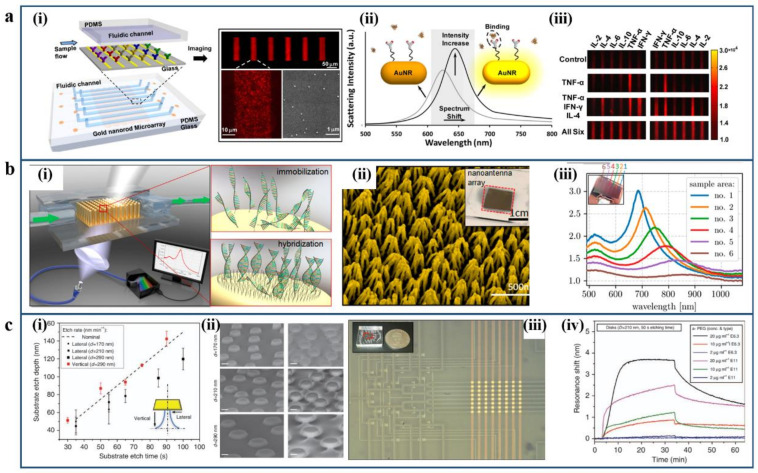
Examples of nanoparticle array-based plasmonic sensing platforms. (**a**) LSPR biosensor with microfluidic patterning and gold nanorods (Au NRs) conjugated with antibodies: (**i**) Schematic of the LSPR microarray chip. (**ii**) The principle of the Au NRs microarray LSPR sensing system. (**iii**) Dark-field images of Au NR microarrays for multiplex cytokine detection. Reproduced with permission from [91]. Copyright 2015 American Chemical Society. (**b**) Vertical arrays of gold nanoantennae-based plasmonic biosensor: (**i**) Schematic of vertical arrays of a gold nanoantennae-based plasmonic biosensor, which is incorporated into a microfluidic chip system. (**ii**) SEM image of vertically aligned nanoantennae. (**iii**) Extinction spectra of vertically aligned nanoantennae. Reproduced with permission from [92]. Copyright 2018 American Chemical Society. (**c**) The LSPR label-free biosensor chip based on electromagnetic decoupling: (**i**) Etch depths with vertical (red) and lateral (black) of glass for substrates covered with gold nanodisks. (**ii**) The SEM images of an LSPR substrate consists of gold nanodisks centered on narrow SiO2 pillars. (**iii**) The photograph LSPR sensor integrated with PDMS microfluidic device containing 64 sensing sites. (**iii**) (**iv**) Real-time sensorgram comparing the interaction of two types of anti-PEG IgGs with different antibody concentrations. Reproduced with permission from [93]. Copyright 2017 Spring Nature.

One example is an LSPR biosensor device with microfluidic patterning and gold nanorods (Au NRs) conjugated with antibodies for high-throughput detection, as shown in Figure 5a. This device can realize parallel immunoassays of six cytokines detection at lower concentrations down to the pg/mL scale in serum medium. By using the dark-field imaging scanning technique, the scattering light intensity across the ensemble of Au NR microarrays can be measured [91]. Stephanie et al. presented a vertically aligned and densely packed gold nanorods arrays-based plasmonic biosensor integrated with the microfluidic channel, which is for the label-free detection of the immobilization, backfilling, and hybridization of short DNA sequences [92]. As shown in Figure 5b, the investigated biochemical reaction of specific binding the target DNA molecules on nanoantennae can be monitored by the change of the LSPR of plasmonic nanostructure array. In some cases, LSPR sensing devices may contain extremely large regions of dielectric materials, which could induce the adsorption of the analyte. These regions should be carefully blocked to avoid compromising the accuracy of measurements. To address this issue, Srdjan S Aćimović et al. developed vertical arrays of the gold nanoantennae-based plasmonic biosensor, as shown in Figure 5c. Such a kind of substrate exhibits markedly reduced vulnerability to nonspecific substrate adsorption [93]. This approach provides a unique substrate design for label-free LSPR biosensing systems with minimal sample consumption and high-throughput merits.

### 3.2. Nanohole Array and Nanocavity Arrays

The sub-wavelength pattern nanoholes or cavities array are usually fabricated on optically thick metal films, and the dimension of the periodic structure is less than the wavelength of incident light propagating through it [94]. The excitation of plasmons of nanoholes by grating coupling of incident light can strongly enhance the EM field near the holes for the EOT spectrum [81]. The pattern nanoholes or cavities array-based plasmonic biosensors can detect a shift of wavelength by transmission spectrum due to the changes of local RI at the plasmonic sensor surface, which is similar to the SPR sensing method [95]. Furthermore, it can also achieve high-throughput multiplexing analysis with high sensitivity when integrated into microfluidic devices. Various kinds of pattern nanoholes or cavities array-based plasmonic biosensors have been developed and integrated with the microfluidic system for biological and biomedical-related assay [96,97,98,99,100].

**Figure 6:**
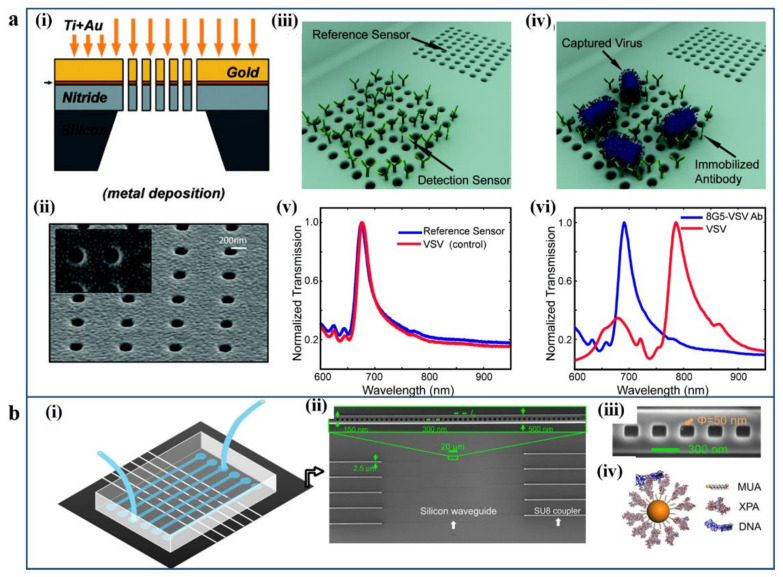
Examples of nanoholes or cavity array-based plasmonic sensing platforms. (**a**) Label-free pattern nanoholes array based in an optofluidic device for the direct detection of live viruses. (**i**) Schematic of plasmonic nanohole arrays fabricated by metal deposition and (**ii**) SEM image of nanohole array sensor. (**iii**,**iv**) The schematic illustration and (**v**,**vi**) the EOT transmission spectrum of antibody functionalized nanohole array biosensor. Reproduced with permission from [101]. Copyright 2010 American Chemical Society. (**b**) A hybrid photonic–plasmonic antenna-in-a-nanocavity biosensor for single-molecule DNA–protein dynamics detection. (**i**) Schematic illustration of the hybrid biosensing system. (**ii**) SEM image of the silicon photonic chip with multiplexed photonic crystal nanobeam cavities and connected by waveguiding components. (**iii**) SEM image of the photonic crystal nanobeam cavity. (**iv**) Illustration of the biofunctionalized gold nanoparticle. Reproduced with permission from [102]. Copyright 2017, American Association for the Advancement of Science.

For instance, Altug’s lab developed label-free pattern nanoholes array-based optofluidic device that can directly detect intact viruses from biological media [101]. As shown in Figure 6a, the sensing platform can utilize group-specific antibodies for highly divergent strains of rapidly evolving viruses based on the EOT effect in plasmonic nanoholes. Such kinds of plasmonic nanohole arrays biosensors can detect live pseudo-Ebola viruses. The effective RI of the medium increases when target analytes bind on the surface of the gold nanostructure, which results in a redshift in the EOT spectrum (Figure 6a(iv)). The sensing platform can quantify the virus’s concentration by immobilized antiviral immunoglobulins for specific capturing. Furthermore, this system is capable of direct detection of different types of viruses via the quantitative refractometric biosensing method. Another example is a hybrid photonic–plasmonic antenna-in-a-nanocavity biosensor for single-molecule DNA–protein dynamics detection without using fluorescent labels, as shown in Figure 6b [102]. The antenna-in-a-nanocavity hybrid system can improve the detection figure of merit and achieve millisecond time resolution. Furthermore, it can realize multiple binding collections in a single measurement.

## 4. State-of-the-Art Sensing Approaches of Microfluidics-Based Plasmonic Biosensors

Owing to the low cost and ease of integration of the microfluidic technique, the plasmonic biosensor can be miniaturized and utilized for detection and assay in bio-related fields. Enormous progress has been made to offer microfluidic plasmonic biosensors that are user-friendly, automatized, and portable instruments from the proof-of-concept laboratory demonstration toward commercially available products that meet special requirements of clinical practice and point-of-care applications. Due to the burgeoning development of micro/nano-optics, there are gradually appearing more advances in plasmon resonance techniques and optical components. In addition to the simplest SPR/LSPR sensing approach that is based on RI change binding measurements, various plasmonic biosensing approaches combined with other advanced techniques have emerged, such as SPR imaging, SERS, and multiplex sensors integration. Hence, we provide a brief overview of the current state-of-the-art microfluidics-based plasmonic biosensing approaches.

### 4.1. SPR/LSPR Sensing Systems

**Figure 7:**
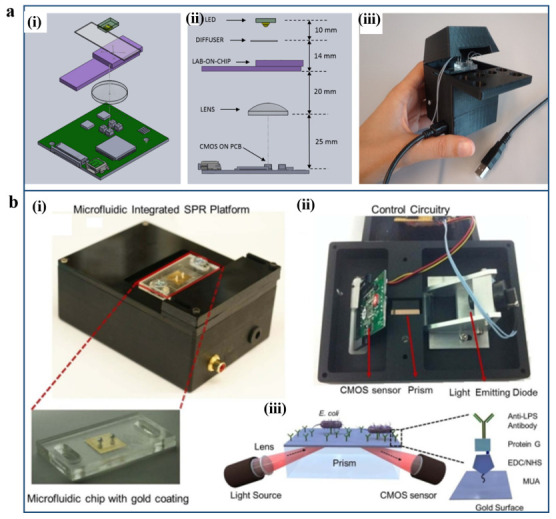
(**a**) Custom-made T-LSPR biosensing device. (**i**) Scheme of the different components in the T-LSPR setup. (**ii**) Side view of the T-LSPR setup with relative distances indicated. (**iii**) Photograph of the complete T-LSPR setup. Reproduced with permission from [105]. Copyright 2015 American Chemical Society. (**b**) Portable multiplex microfluidic-integrated SPR biosensing platform for detection of the pathogen. (**i**) The photograph of the top side of the device was mounted with surface-activated disposable microfluidic chips. (**ii**) The electronic setup of the device is represented from the bottom. (**iii**) Scheme illustration of the multiplex microfluidic-integrated SPR biosensing system. Reproduced with permission from [106]. Copyright 2015 Spring Nature.

The principle and related example-based SPR/LSPR biosensing systems have been introduced in Section 3 in detail. Hence, some alternative plasmonic sensing systems integrated with microfluidic work will be represented here. In addition to the extinction measurement, LSPR can also generate a non-propagating plasmon mode when employed on a disconnected pattern of the thin metal film, which is called transmission configuration (T-LSPR) [103]. The T-LSPR can be readily characterized from the transmitted when it is excited by the passing-through light, which can greatly simplify the detection system [104]. One example is a portable, palm-sized T-LSPR device developed by Carlotta Guiducci’s group. Such a T-LSPR sensing device contains off-the-shelf components, which have coupled with DNA-based aptamers specific to the antibiotic tobramycin, as shown in Figure 7a [105]. The key part of the T-LSPR setup is the aptamer-functionalized gold nanoislands (NIs) sensing chip, which is deposited on a glass slide and covered with fluorine-doped tin oxide (FTO). The LSPR of gold NIs in the visible range wavelength can match the sensitivity of the light detector, which consists of the complementary metal–oxide semiconductor (CMOS) image sensor. With this T-LSPR sensing mode, it can realize real-time label-free detection of tobramycin in buffer down to 0.5 μM concentration. 

Another example is a portable and multiplex microfluidic-integrated SPR biosensing platform for the detection of the pathogen. Tokel et al. have demonstrated that their inexpensive microfluidic-integrated SPR sensing platform can be quantified to detect two types of bacteria rapidly, as shown in Figure 7b [106]. The platform presented reliable capture and detection of *E. coli* and *S. aureus* in a large range of concentrations in different buffer solutions with multiplexing and specificity capability. Such an integrated system can potentially be generalized to other well-defined biomarkers such as pathogens or for immunodiagnostics for the targeted applications.

### 4.2. Surface Plasmon Resonance Imaging (SPRi)

The multiplexed analysis of plasmonic biosensors is an important challenge for their application in the clinical field. The simplest way to reach high-throughput detection can be provided by SPR Imaging (SPRi), which was first introduced by Benno Rothenhäusler and Wolfgang Knoll in 1988 [107]. SPRi is an optical technique, which can spatially monitor localized differences in the reflectivity of incident light caused by interactions with analyte via prism coupling [108]. In this technique, the excitation of the SPR in an extended area can be realized by a collimated monochromatic light beam via prism coupling. Then, the intensity variations of the reflected light due to RI changes can be analyzed in a 2D charge-coupled device (CCD) camera. Multi-analysis can be implemented by evaluations of the 2D reflected intensity pattern when various analytes are immobilized at different areas at the arrayed surface. The signal detected by the CCD camera, such as SPR images of the arrayed surface and sensorgrams (resonance signal vs. time), allow simultaneous analysis of the interactions in high-throughput ways without any labeling [109]. Furthermore, tremendous developments and improvements have been made in the SPRi technique-based biosensing detection system. In addition to reflected intensity, the angular, wavelength, phase, and polarization interrogation with incident light-based, SPRi sensors have also been exploited in recent years [110,111,112,113,114,115].

**Figure 8:**
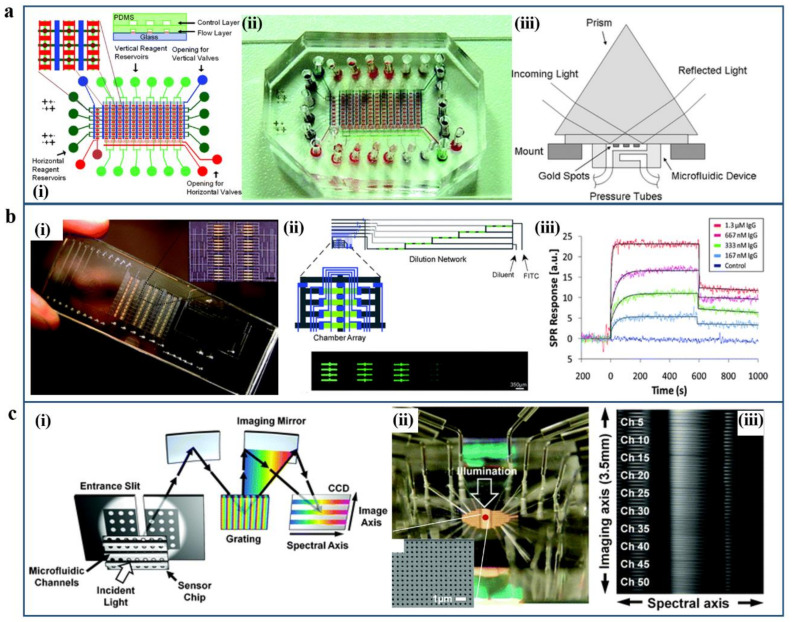
Examples of SPRi-based plasmonic sensing platforms. (**a**) Immunoassays by microfluidic-integrated SPRi sensing device. (**i**) The layout of the microfluidic device. (**ii**) Visualization photographs consist of valves and flow channels in the microchip device. (**iii**) The schematic illustration of the setup for a microfluidics-based SPR imaging device. Reproduced with permission from [113]. Copyright 2008 Royal Society of Chemistry. (**b**) Parallel microfluidic surface plasmon resonance imaging arrays. (**i**) Photograph of the microfluidic chip bonded to a gold-patterned glass slide. (**ii**,**iii**) Schematic diagram and fluorescence images of the serial dilution network and the chaotic advection micromixers on the chip. Reproduced with permission from [114]. Copyright 2010 Royal Society of Chemistry. (**c**) Fifty microfluidic channels-based real-time full-spectral SPRi sensing platform for affinity measurements. (**i**) Schematic illustration of the spectral imaging system setup. (**ii**) Photograph of inlet and outlet ports of the microfluidic chip; inset is the SEM image of an area of the nanohole chip. (**iii**) Fifty parallel channels of full spectral content imaging. Reproduced with permission from [115]. Copyright 2012 Royal Society of Chemistry.

Meanwhile, the microfluidic-assisted SPRi biosensing system can offer real-time monitoring with small sample consumption in high-through channels [116,117,118,119,120,121]. Zare et al. have developed an SPRi-based microfluidic sensing device containing an array of gold spots, as shown in Figure 8a [113]. The antibody–antigen recognition and binding immunoreaction can be detected and monitored by the surface plasmon resonance (SPR) imaging technique. Hence, the sensitivity of such a device can reach the sub-nanomolar level, and the immunoreaction could be detected and quantitatively characterized in about 10 min. Ouellet et al. presented a microchip device using soft lithography techniques to integrate a parallel microfluidic array with a high-throughput SPRi technique to detect the binding affinities of antibodies against the protein [114]. This contained 264 element-addressable chambers with an on-chip parallel array device that can realize interrogating binding events of 264 different immobilized ligands against multiple analytes in a single experiment, as shown in Figure 8b. The interrogation of multiple ligands to multiple analytes simultaneously demonstrates the versatility accommodation of such a kind of SPRi biosensing device. A real-time full-spectral SPR imaging system based on large-area metallic nanohole arrays has been developed by the Sang-Hyun Oh group [115]. This device has an imaging spectrometer with a cooled CCD camera, which can couple with a microfluidic chip with 50 parallel channels. As shown in Figure 8c, the receptor–ligand binding kinetics affinities can be extracted and transformed to high-throughput full-spectral imaging and produced accurate and quantified measurement by the SPRi instrument simultaneously. This multi-channel imaging sensing system allows a wide range of bioanalytical research and high-throughput proteomics, which is beneficial to acquire real-time acquisition of full spectra SPRi assays.

### 4.3. Surface-Enhanced Raman Scattering

As one of the most well-known practical applications of the plasmonic coupling effect, SERS can combine the laser spectroscopy technique with the unique SPR and LSPR properties of noble metal nanostructures [122]. The SERS technology can amplify the vibrational spectroscopic signals of Raman and offer high-level structural information of target molecules adsorbed on the surface of plasmonic nanostructures [123]. Hence, SERS technology can offer ultrasensitive detection limits with plasmonic enhancement. This characteristic allows the trace detection of biomarkers with their structural identity down to the single-molecule level. SERS is a promising sensing method for fast and reliable biomarker identification and analytics in biomedical diagnosis fields [124]. The SERS sensors can be classified into two different types according to the sources of SERS signals. One type is target molecules that can be directly detected by recognizing their characteristic fingerprint in Raman spectra. On the other hand, another type needs the surface functionalization step to build specific molecular conjugation between target analytes with surface-functionalized materials [125]. Furthermore, SERS can also combine with different microfluidic approaches for sensitive optofluidic detection and analysis [90,126,127]. In SERS-based microfluidic systems, the analytes can be controlled and analyzed in microchannels, because the analytes can fully interact and be attached to a plasmonic nanostructured-based sensor substrate [128]. Special topographical morphologies of the SERS-active plasmonic substrate contained in the microfluidic chip could influence the detection performances [90,126,128].

**Figure 9:**
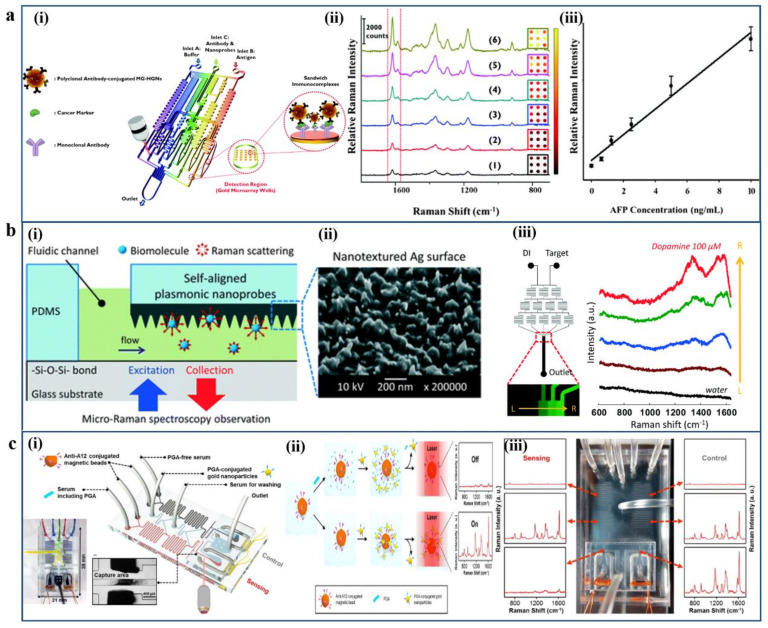
Examples of SERS-based microfluidic biosensing platforms. (**a**) SERS-based immunoassay with gold-patterned microarray wells-embedded gradient microfluidic chip: (**i**) Schematic illustration of gold-patterned microarray wells-embedded gradient microfluidic chip (**ii**,**iii**) SERS-based quantitative immunoassay for alpha-fetoprotein (AFP) model protein marker AFP antigen. Reproduced with permission from [129]. Copyright 2012 Royal Society of Chemistry. (**b**) Optofluidic SERS chip with self-aligned plasmonic nanoprobes along microfluidic channels. (**i**) Schematic illustration of the optofluidic SERS chip. (**ii**) SEM image of plasmonic nanoprobes self-aligned along microfluidic channels. (**iii**) Optofluidic SERS detection of dopamine molecules. Reproduced with permission from [130]. Copyright 2013 Royal Society of Chemistry. (**c**) SERS-based solenoid microfluidic sensor for anthrax biomarker detection. (**i**) Schematic illustration of SERS-based solenoid-embedded dual-channel microfluidic sensor for competitive immunoassay. (**ii**) Schematic illustration of the principle of the PGA marker competitive immunoassay in serum by SERS spectra. (**iii**) Photograph of the solenoid-embedded microfluidic chip and SERS spectra at six different channel positions. Reproduced with permission from [131]. Copyright 2015 Elsevier.

For example, Choo et al. have developed a SERS-based immunoassay with a gold-patterned microarray wells-embedded gradient microfluidic chip, which can realize programmable and fully automatic assay, as shown in Figure 9a [129]. Such a kind of gold microarray wells-embedded gradient microfluidic channel design can offer a convenient and reproducible SERS-based quantitative immunoassay platform for cancer biomarkers such as alpha-fetoprotein (AFP) model protein marker AFP antigen. Furthermore, the N cascade-mixing stage microfluidic gradient generators can eliminate the tedious manual dilution process, and the total assay time from serial dilution to SERS detection takes less than 60 min. An optofluidic SERS chip with self-aligned plasmonic nanoprobes along microfluidic channels is presented and shown in Figure 9b. 

The plasmonic nanoprobes are selectively patterned on the PDMS microfluidic channel walls. The plasmonic nanoprobes can generate plentiful hot spots for the Raman enhancement in microfluidic networks, which can gain successful SERS detection of dopamine molecules [106]. This optofluidic SERS sensing platform can be readily expanded with diverse functions for advanced biomedical-related assays. A SERS-based solenoid microfluidic sensor has been reported for anthrax biomarker detection by Choo et al., as shown in Figure 9c [131]. This fully automated SERS-based solenoid-embedded microfluidic device can realize fast, sensitive, quantitative detection of anthrax biomarker poly-γ-D-glutamic acid (PGA). The SERS signals can be directly measured and analyzed through the competitive reaction between PGA and PGA-conjugated gold nanoparticles with anti-PGA-immobilized magnetic beads in a microfluidic environment. The limit of detection of PGA in serum can reach up to 100 pg/mL in such an SERS-based microfluidic sensor.

### 4.4. Multiplex Sensors Integration

In addition to the above plasmonic biosensing techniques as analytical platforms for the aforementioned bio-related applications, the use of plasmonic biosensors as analytical platforms has not been fully accomplished. There are also reports concentrating on the combination of multiple sensors based on different principles. Such kinds of multiplex sensors integration can offer more portable and powerful chip devices for potential clinical and biomedical applications [35,132].

**Figure 10:**
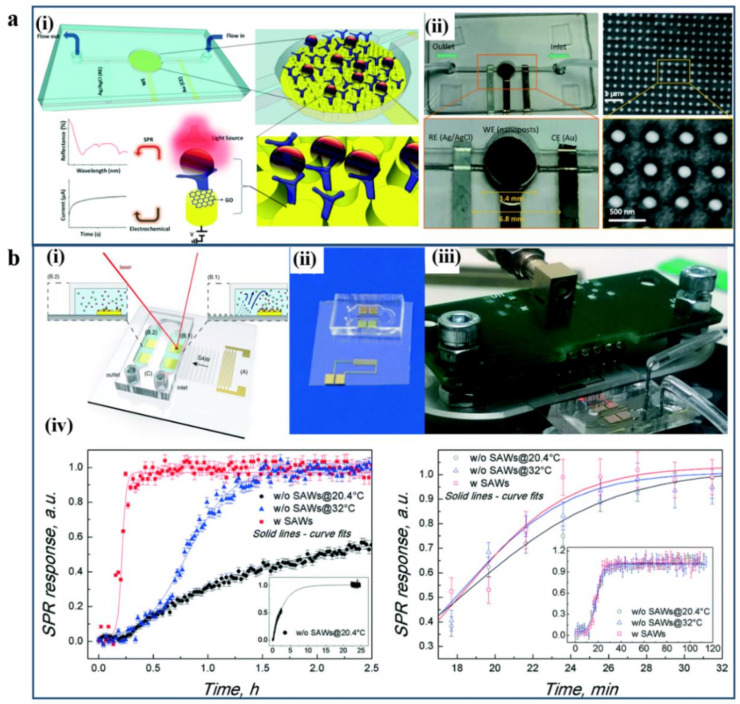
(**a**) Integrated dual-modality microfluidic sensor: (**i**) Schematic of the integrated dual-modality microfluidic biosensor chip for the detection of cancer biomarkers. (**ii**) The photograph of the fabricated integrated dual-modality sensor chip and the SEM image for a patterned periodic array of nanoposts. Reproduced with permission from [133]. Copyright 2018 Royal Society of Chemistry. (**b**) SAW-enhanced grating coupling phase-interrogation SPR microfluidic biosensor. (**i**) Schematic illustration of the SAW-enhanced grating coupling phase-interrogation SPR microfluidic biosensor. (**ii**,**iii**) Configuration for acoustic-plasmonic sensor chip and the SAW-driven SPR detection biosensor setup. (**iv**) SPR responses of thiol-polyethylene glycol adsorption and avidin/biotin binding kinetics under three different experimental conditions. Reproduced with permission from [134]. Copyright 2016 Royal Society of Chemistry.

For example, an integrated dual-modality microfluidic sensor chip has been developed by Liang Dong et al. for target biomarker molecules detection, as shown in Figure 10a [132]. The device consists of a patterned periodic array of gold nanoposts functionalized with graphene oxide nanosheets and antibodies. The integrated dual-modality sensor can operate simultaneously and coupling incident light and voltage sources in a single nanopost. 

Therefore, the generated SPR and electrochemical signals can implement higher sensitivity as well as the dynamic measurements of antigen–antibody bindings. Another example is a surface acoustic wave (SAW)-enhanced SPR microfluidic biosensor developed by Cecchini et al., as shown in Figure 10b [134]. The phase-interrogation grating-coupling SPR and SAW-induced mixing are integrated into a single lithium niobate microchip device. By exploiting the high sensitivity of grating-coupling SPR under azimuthal control, the thiol-polyethylene glycol adsorption and avidin/biotin binding kinetics can be monitored. This SAW-induced integrated SPR biosensing microchip can significantly reduce the bioreactions experiment duration. Hence, such implementation of a nanostructured SAW–SPR microfluidic biosensor can offer significant improvement of the molecule binding kinetics on a single, portable device.

## 5. Conclusions and Outlook

In this review, we have summarized and proven that the microfluidic-integrated plasmonic biosensor can be an advanced detection tool for various biomolecule interactions with different types of plasmonic structure-based sensing systems. It reveals that the real-time ultrasensitivity and high throughput of various biomolecules can be realized by combining the advantages of plasmonic biosensors with microfluidic chip platforms. We believe that the recent developments and the summary of literature work will give guides to discover different strategies toward more optimized plasmonic biosensor devices for potential biological, biomedical, and diagnostics applications. With the development and advancement of microfluidics, nanofabrication technologies, and portable instrumentation systems, it can be predicted that the microfluidic-integrated plasmonic biosensor will become an ideal platform for greater sensitivity, more miniaturization, and even lower cost with a multiplexed analytes signal process at the same time. 

Although the potential for microfluidic-integrated plasmonic techniques has greatly impacted biosensing applications, there still exist several challenges and limitations. The advantages and drawbacks of the discussed different sensing approaches of plasmonic biosensors in the microfluidic system are shown in Table 1. For example, the SPRi-based biosensor has faced drawbacks in the resolution due to the channel cross-talk, which limits ultrasensitive detection. Although the anisotropic plasmonic microarray can improve the sensitivity and limit of detection of desired analytes, the device could not sustain accuracy after several times of utilization due to the contamination and corrosion of analytes and media substances. So, one key challenge is to increase the stability of the plasmonic sensor. The surface biofunctionalization of plasmonic structures is also a vital part to enhance the biosensor performance and guarantee more reliable and accurate detection and analysis of analytes in a real clinical scenario with various types of bio/chem molecules. Another challenge is that it may be difficult to integrate all the components into a single portable platform due to technical problems for miniaturized optical-related instrument parts, such as illumination systems and spectrometers, for potential clinic and point-of-care applications.

Therefore, microfluidic plasmonic biosensors have made efforts devoted to the integration of multiplexor hybrid sensors with other functionalities. The advanced development in the fields of microelectromechanical fabrication systems such as 3D/4D high precisely printing techniques for sensor microfluidics and printing will also nutritious plasmonic biosensor technology. As for materials science study, the emergence of new unique properties of plasmonic materials will also immensely benefit developing new intriguing multiplex microfluidics-based plasmonic biosensors with more robust and stable performance with a relatively inexpensive fee. Continuous effort and improvements in multidisciplinary fields can give rise to more excellent performance for the plasmonic biosensor technologies. Furthermore, combining with the irreplaceable advantage of the microfluidic chip including integration, miniaturization, and portability, it will be gradually increasing the trend in the utility of microfluidics-based plasmonic biosensors for point-of-care, smart, and potable clinic detection and diagnostics applications in the future.

## Figures and Tables

**Table 1:** The comparison of different sensing approaches of plasmonic biosensors in the microfluidic system.

SENSING METHOD	ADVANTAGES	DRAWBACKS
**SPR/LSPR sensing**	Label-free, simple setup	Susceptible to environmental disturbance
**SPR imaging**	Label-free, multi-channel up to 50	Channel cross-talk with low resolution
**SERS**	High-sensitive, selectivity with different species of biomarker	Equipment expensive
**Multiplex integration**	Reduce the bioreactions duration and enhance molecular binding	Complex system set up
